# Crystal structures of (*R*_S_)-*N*-[(1*R*,2*S*)-2-benz­yloxy-1-(2,6-di­methyl­phen­yl)prop­yl]-2-methyl­propane-2-sulfinamide and (*R*_S_)-*N*-[(1*S*,2*R*)-2-benz­yloxy-1-(2,4,6-tri­methyl­phen­yl)prop­yl]-2-methyl­propane-2-sulfinamide: two related protected 1,2-amino alcohols

**DOI:** 10.1107/S1600536814022570

**Published:** 2014-10-24

**Authors:** Matthew R. Carbone, Garrick A. Centola, Adam Haas, Kevin P. McClelland, Michael D. Moskowitz, Angelo M. Verderame, Mikael S. Olezeski, Louis J. Papa, Stephanie C. M. Dorn, William W. Brennessel, Daniel J. Weix

**Affiliations:** a Department of Chemistry, 120 Trustee Road, 412 Hutchison Hall, University of Rochester, Rochester, NY 14627, USA

**Keywords:** crystal structure, sulfinamide, diastereomer, amino alcohol, hydrogen bonding, NMR, column chromatography

## Abstract

The title compounds, C_22_H_31_NO_2_S, (1), and C_23_H_33_NO_2_S, (2), are related protected 1,2-amino alcohols. They differ in the substituents on the benzene ring, *viz.* 2,6-di­methyl­phenyl in (1) and 2,4,6-tri­methyl­phenyl in (2). The plane of the phenyl ring is inclined to that of the benzene ring by 28.52 (7)° in (1) and by 44.65 (19)° in (2). In the crystal of (1), N—H⋯O=S and C—H⋯O=S hydrogen bonds link mol­ecules, forming chains along [100], while in (2), similar hydrogen bonds link mol­ecules into chains along [010]. The absolute structures of both compounds were determined by resonance scattering.

## Abstract 2

Mol­ecules of two related 1,2-amino alcohols are linked *via* N—H⋯O=S hydrogen bonds, forming chains along [100] for the first compound and along [010] for the second compound.

## Chemical context   

1,2-Amino alcohols are found in a variety of pharmaceutically active compounds (Lee & Kang, 2004 ▶) and have been used extensively as components of chiral ligands and auxiliaries in asymmetric synthesis (Ager *et al.*, 1996 ▶; Pu & Yu, 2001 ▶). In order to develop new chiral ligands and as part of an advanced undergraduate laboratory course, we sought to make a series of 2-aryl-1-methyl-1,2-amino alcohols. The most straightforward synthesis of these compounds was reported by Ellman (Tang *et al.*, 2001 ▶; Evans & Ellman, 2003 ▶). The method relies upon the chiral ammonia equivalent, 2-methyl-2-propane­sulfinamide (*tert*-butane­sulfinamide), which is readily available from a variety of commercial sources or easily synthesized on scale (Weix *et al.*, 2005 ▶). In the original Ellman report, the absolute configuration of the products was determined by deprotection of the amine and alcohol, cyclization to form the corresponding oxazolidinone, and correlation of the ^1^H NMR spectra with the literature (Zietlow & Steckhan, 1994 ▶).
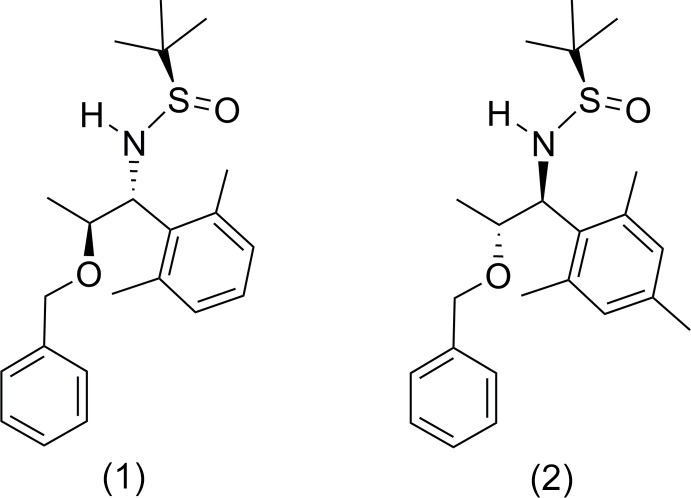


**Figure 1:**
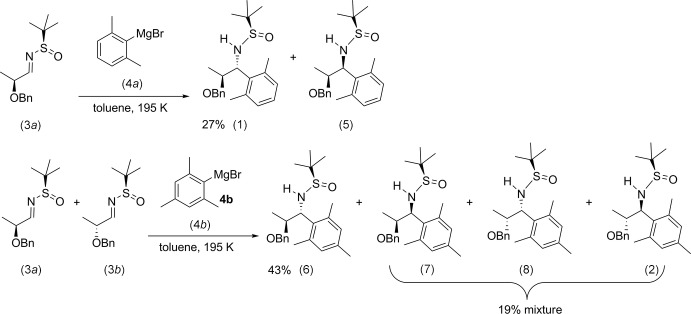
(Top) Reaction scheme depicting the synthesis of (1) and (5) from (3*a*), for which (1) is the major product of the reaction. (Bottom) Reaction scheme depicting the synthesis of (6) and (7) from (3*a*), and (8) and (2) from (3*b*), for which (6) is the major product of the reaction from (3*a*), and (8) is the major product from (3*b*).

We report herein on the syntheses and structures of two different but related protected 1,2-amino alcohols, (1) and (2), from the addition of an aryl­magnesium bromide to an *N*-*tert*-butane­sulfinyl imine (Evans & Ellman, 2003 ▶). The reaction of imine (3*a*) with xylylmagnesium bromide, (4*a*), (see Fig. 1 ▶) resulted in a mixture of amino alcohol products from which the major product of the reaction, (1), was isolated in 27% yield after chromatographic separation of the diastereomers. The stereochemistry of this major product was confirmed by X-ray diffraction and the result is consistent with the sense of induction reported by Evans & Ellman (2003 ▶).

The analogous reaction with mesitylmagnesium bromide, (4*b*), also resulted in a mixture of products, from which the major product, (6), was isolated in 43% yield. A mixture of other diastereomers was also isolated, from which a crystal suitable for X-ray diffraction was grown. Unexpectedly, X-ray analysis showed this crystal to be (2), a product that could only have derived from a diastereomerically different isomer of (3*a*). Upon further investigation, we discovered that the starting material, which we had assumed was pure (3*a*), contained the minor diastereomer, (3*b*), in about 8% (determined by ^1^H NMR; Fontenelle *et al.*, 2014 ▶), which had formed due to racemization in the synthesis of (3*a*). Based on the work of Evans & Ellman (2003 ▶), it was deduced that (2) is the *minor* product expected from the reaction of (3*b*) with an aryl­magnesium bromide. Although no further separations were performed on this mixture that contained (2), it follows that the other diastereomers present were (7), the minor product from the reaction with (3*a*), and (8), the major product from the reaction with the slight impurity of (3*b*).

## Structural commentary   

**Figure 2:**
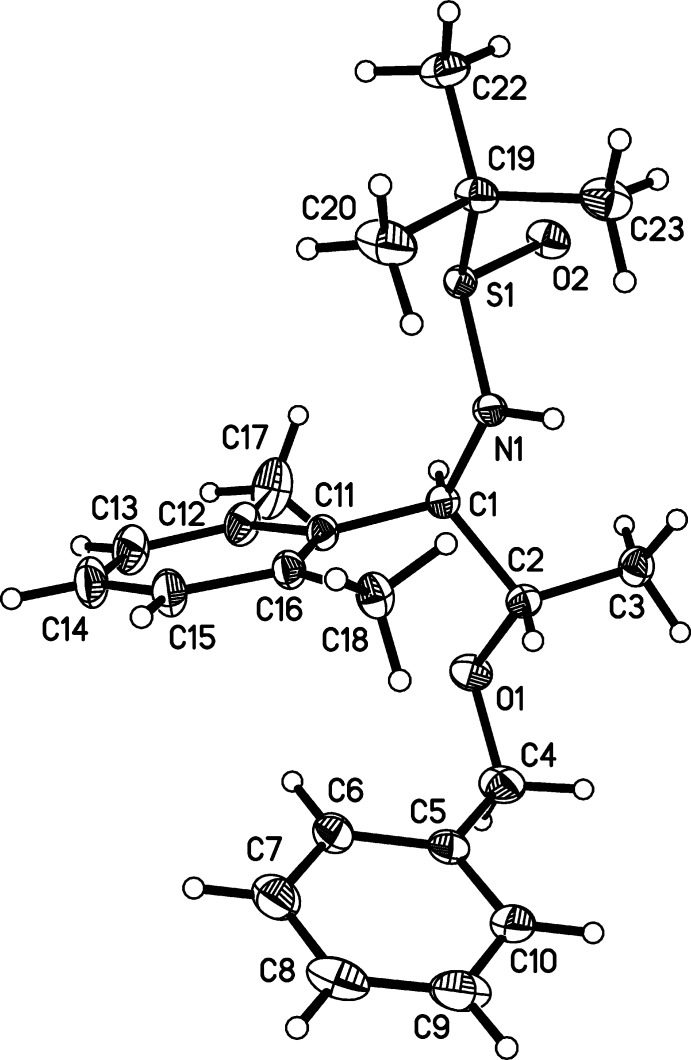
The mol­ecular structure of compound (1), with atom labelling. Displacement ellipsoids are drawn at the 50% probability level.

The mol­ecular structures of compounds (1) and (2) are illus­trated in Figs. 2 ▶ and 3 ▶, respectively. The essential difference in the conformation of the two compounds is that the phenyl ring (C5–C10) is inclined to the benzene ring (C11–C16) by 28.52 (7)° in (1) and by 44.65 (19)° in (2).

## Supra­molecular features   

**Figure 4:**
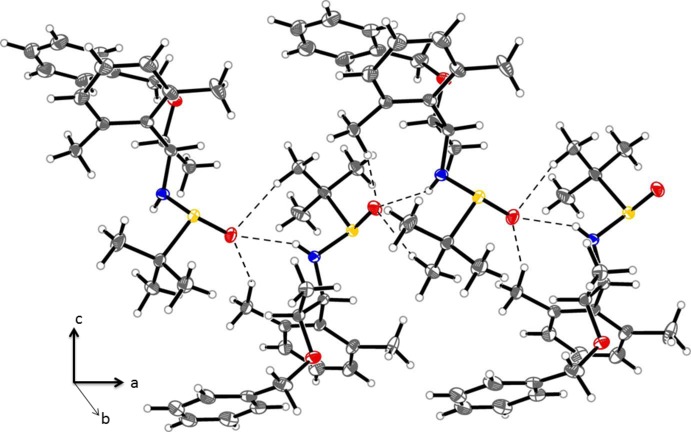
A partial view of the crystal packing of compound (1), illustrating the formation of the hydrogen-bonded chains along [100] (hydrogen bonds are shown as dashed lines; see Table 1 ▶ for details). Displacement ellipsoids are drawn at the 50% probability level.

**Figure 5:**
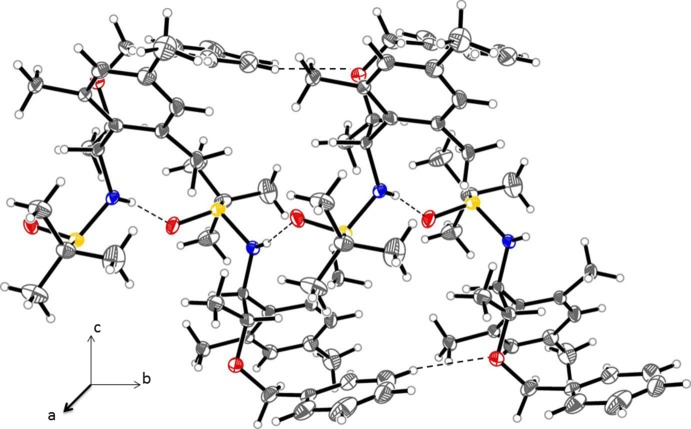
A partial view of the crystal packing of compound (2), illustrating the formation of the hydrogen-bonded chains along [010] (hydrogen bonds are shown as dashed lines; see Table 2 ▶ for details). Displacement ellipsoids are drawn at the 50% probability level.

In the crystals of both (1) and (2), chains are formed *via* inter­molecular hydrogen bonding (Tables 1 ▶ and 2 ▶). In (1), mol­ecules are linked along the [100] direction by a combination of classical (N—H⋯O=S) and non-classical (C—H⋯O=S) hydrogen bonds (Table 1 ▶ and Fig. 4 ▶). In (2), mol­ecules are linked along the [010] direction also by classical (N—H⋯O=S) and non-classical (C—H⋯O=S) hydrogen bonds (Table 2 ▶ and Fig. 5 ▶).

## Database survey   

Although there are 78 structures of *N*-sulfinyl-protected 1,2-amino alcohols in the Cambridge Structural Database (CSD, Version 5.35, last update May 2014; Groom & Allen, 2014 ▶), only seven of these structures have substitution at the 1-position and an aryl group at the 2-position. Of these compounds, only three have a *tert*-butane­sulfinyl group [CSD refcodes CAVQOG (Zhong *et al.*, 2005 ▶), FIZBIB (Jiang *et al.*, 2014 ▶) and WOBNEI (Buesking & Ellman, 2014 ▶)], and the other four contain *p*-toluene­sulfinyl groups [CSD refcodes PAQZIR (Zhao *et al.*, 2005 ▶), RUXZUG (Ghorai *et al.*, 2010 ▶), WADYOR (Fadlalla *et al.*, 2010 ▶) and SICSII (Guo *et al.*, 2012 ▶)]. However, none of these seven compounds were synthesized by our method of inter­est.

## Synthesis and crystallization   

The starting sulfinamide, (*R*,*E*)-*N*-(2-(benz­yloxy)propyl­idene)-2-methyl­propane-2-sulfinamide, (3*a*), was prepared from *S*-ethyl lactate (Enders *et al.*, 2002 ▶; Evans & Ellman, 2003 ▶). Grignard reagents (4*a*) and (4*b*) were prepared from 2-bromoxylene and 2-bromo­mesitylene, respectively (Tilstam & Weinmann, 2002 ▶). The synthesis of the title compounds is illustrated in Fig. 1 ▶.

**General procedure**

To an oven-dried 50 ml Schlenk flask equipped with a magnetic stirrer bar and a rubber septum, sulfinamide (3*a*) and toluene (20 ml) were added and the mixture was cooled to 195 K under nitro­gen. The Grignard reagent (4*a*) or (4*b*) in toluene was placed under positive nitro­gen pressure and was added to the Schlenk flask dropwise by cannula at 195 K. The reaction was stirred at 195 K and stopped when complete consumption of the imine was confirmed by thin-layer chromatography (30% ethyl acetate in hexa­nes, stained with ceric ammonium molybdate). The reaction was quenched with aqueous saturated sodium sulfate (1.5 ml), then the mixture was warmed to room temperature, dried over sodium sulfate, filtered through Celite, and the solvent was removed under reduced pressure. The ratio of diastereomers was determined by ^1^H NMR of the crude material, specifically by examining the amine (N—H) proton resonances. The chemical shifts of *anti* diastereomers like (1) and (6) were found around δ = 3.78 p.p.m., while those for *syn* diastereomers were found slightly further upfield at δ = 3.61 (mixture, see below) and 3.66 (5) p.p.m.. The crude viscous yellow oil was purified by column chromatography. Crystals suitable for single-crystal X-ray diffraction were obtained from slow evaporation of methanol solutions.

**(** ***R*** **_S_)-** ***N*** **-[(1** ***R*** **,2** ***S*** **)-2-benz­yloxy-1-(2,6-di­methyl­phenyl)propyl]-2-methyl­propane-2-sulfinamide (1):**

The reaction of sulfinamide (3*a*) (0.631 g, 2.36 mmol) with xylylmagnesium bromide [(4*a*), 3.80 equiv, 8.87 mmol], performed according to the general procedure, yielded a 2.5:1 ratio of diastereomers, (1) to (5), respectively (see Fig. 1 ▶). The light-yellow oil was purified by column chromatography (100% diethyl ether) to yield a light-yellow solid (239 mg, 27%).

**(1)**: m.p.: 346–348 K, ^1^H NMR (500 MHz, CDCl_3_): δ 1.20 (*d*, *J* = 0.3, 9H), 1.32 (*d*, *J* = 6.1, 3H), 2.36 (*s*, 3H), 2.43 (*s*, 3H), 3.71–3.70 (*m*, 1H), 3.99 (*td*, *J* = 6.7, 0.3, 1H), 4.27 (*d*, *J* = 11.8, 1H), 4.39 (*d*, *J* = 11.8, 1H), 4.92–4.89 (*m*, 1H), 6.96–6.94 (*m*, 1H), 7.02–7.01 (*m*, 3H), 7.08 (*d*, *J* = 7.6, 1H), 7.22 (*d*, *J* = 4.6, 3H). ^13^C NMR (126 MHz, CDCl_3_): δ 17.65, 21.62, 21.77, 22.71, 55.48, 59.01, 71.27, 76.41, 127.49, 127.60, 127.85, 128.35, 128.50, 130.43, 134.91, 137.22, 138.32, 138.57. IR (neat): 3271, 1084, 1041 cm^−1^. Analysis calculated for C_22_H_31_NO_2_S (%), 70.74 C, 8.36 H, 3.75 N, found (%) 70.99 C, 8.58 H, 3.66 N.

**(** ***R*** **_S_)-** ***N*** **-[(1** ***S*** **,2** ***R*** **)-2-benz­yloxy-1-(2,4,6-tri­methyl­phenyl)propyl]-2-methyl­propane-2-sulfinamide (2)**:

The reaction of sulfinamide (3*a*) (0.757 g, 2.83 mmol), which contained an impurity (8%) of sulfinamide (3*b*), with mesitylmagnesium bromide [(4*b*), 3.00 equiv, 8.50 mmol] in toluene, performed according to the general procedure, yielded a mixture of *anti* and *syn* diastereomers. The light-yellow oil was purified by column chromatography (80% diethyl ether in hexa­nes) to yield two white solids. The first was the expected major product (6) (467 mg, 43%). The second (207 mg, 19%) was determined to be a mixture of diastereomers (based on ^1^H NMR) that contained (2) (confirmed by X-ray crystallography) and two others, likely (7) and (8) (see Fig. 1 ▶). No further characterization or separation was performed on this mixture.

**(6)**: ^1^H NMR (500 MHz, CDCl_3_): δ 1.17 (*s*, 9H), 1.29 (*d*, *J* = 6.1, 3H), 2.26 (*s*, 3H), 2.33 (*s*, 3H), 2.39 (*s*, 3H), 3.72–3.71 (*m*, 1H), 3.98–3.95 (*m*, 1H), 4.29 (*d*, *J* = 11.9, 1H), 4.39 (*d*, *J* = 11.8, 1H), 4.88–4.86 (*m*, 1H), 6.77 (*s*, 1H), 6.84 (*s*, 1H), 7.06 (*d*, *J* = 4.3, 2H), 7.22 (*s*, 3H). ^13^C NMR (126 MHz, CDCl_3_): δ 17.61, 20.97, 21.56, 21.65, 22.76, 55.44, 58.65, 58.67, 71.30, 76.66, 127.58, 127.88, 128.34, 129.38, 130.80, 131.22, 137.13, 138.45. IR (neat): 3271, 1057 cm^−1^. Analysis calculated for C_23_H_33_NO_2_S (%), 71.27 C, 8.58 H, 3.61 N, found (%) 70.55 C, 8.62 H, 3.49 N.

## Refinement   

Crystal data, data collection and structure refinement details are summarized in Table 3 ▶. For (1), the absolute configuration was determined using 4260 quotients, which gave a Flack parameter of 0.005 (12). The value obtained without *D*_obs_(h) as a restraint was −0.02 (3), calculated from 5203 Friedel pairs. For (2), the absolute configuration was determined using 1713 quotients, which gave a Flack parameter of 0.03 (6). The value obtained without *D*_obs_(h) as a restraint was −0.04 (8), calculated from 2882 Friedel pairs. In (2), the needle-shaped crystal diffracted weakly at higher angles. The cut-off resolution of 0.72 Å was chosen to maximize the number of enanti­omer-determining reflections, while limiting the inclusion of very weak high-angle data. The largest residual peak of 0.72 e Å^−3^ is located in the S1—C20 bond.

For both structures, the amine H atoms were located from difference Fourier maps and freely refined. The C-bound H atoms were placed geometrically and treated as riding with C—H = 0.95–1.00 Å and with *U*_iso(_H) = 1.5*U*_eq_(C) for methyl H atoms and = 1.2*U*_eq_(C) for other H atoms.

## Supplementary Material

Crystal structure: contains datablock(s) 1, 2, global. DOI: 10.1107/S1600536814022570/su2794sup1.cif

Structure factors: contains datablock(s) 1. DOI: 10.1107/S1600536814022570/su27941sup2.hkl

Structure factors: contains datablock(s) 2. DOI: 10.1107/S1600536814022570/su27942sup3.hkl

Supporting information file. DOI: 10.1107/S1600536814022570/su27941sup4.cml

Supporting information file. DOI: 10.1107/S1600536814022570/su27942sup5.cml

CCDC references: 1029177, 1029178

Additional supporting information:  crystallographic information; 3D view; checkCIF report

## Figures and Tables

**Figure 3:**
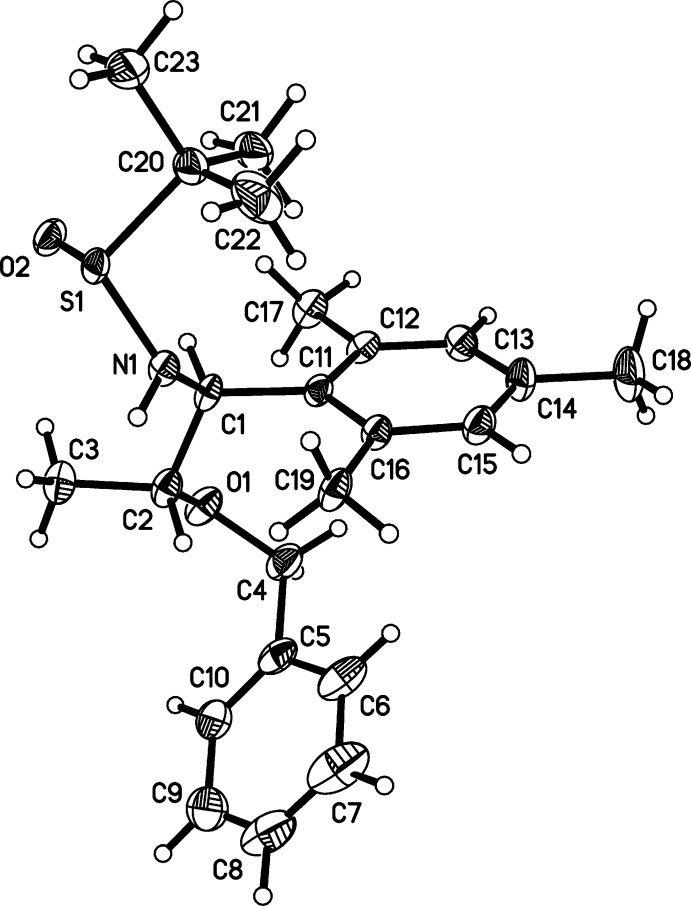
The mol­ecular structure of compound (2), with atom labelling. Displacement ellipsoids are drawn at the 50% probability level.

**Table 1:** Hydrogen-bond geometry (, ) for (1)

*D*H*A*	*D*H	H*A*	*D* *A*	*D*H*A*
N1H1O2^i^	0.84(2)	2.23(2)	3.0039(15)	152.8(7)
C18H18*A*O2^i^	0.98	2.52	3.4077(17)	150
C23H23*B*O2^i^	0.98	2.59	3.5534(17)	167

Symmetry code: (i) 
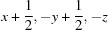
.

**Table 2:** Hydrogen-bond geometry (, ) for (2)

*D*H*A*	*D*H	H*A*	*D* *A*	*D*H*A*
N1H1O2^i^	0.83(4)	2.08(4)	2.890(4)	169(4)
C7H7*A*O1^ii^	0.95	2.59	3.501(6)	160

Symmetry codes: (i) 
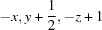
; (ii) 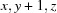.

**Table 3:** Experimental details

	(1)	(2)
Crystal data		
Chemical formula	C_22_H_31_NO_2_S	C_23_H_33_NO_2_S
*M*_r_	373.54	387.56
Crystal system, space group	Orthorhombic, *P*2_1_2_1_2_1_	Monoclinic, *P*2_1_
Temperature (K)	100	100
*a*, *b*, *c* ()	9.1567(13), 10.2951(15), 22.494(3)	10.535(3), 7.984(2), 13.481(4)
, , ()	90, 90, 90	90, 103.519(5), 90
*V* (^3^)	2120.5(5)	1102.5(5)
*Z*	4	2
Radiation type	Mo *K*	Mo *K*
(mm^1^)	0.17	0.16
Crystal size (mm)	0.40 0.25 0.20	0.50 0.14 0.10

Data collection		
Diffractometer	Bruker APEXII CCD	Bruker *SMART* APEXII CCD platform
Absorption correction	Multi-scan (*SADABS*; Bruker, 2014 ▶)	Multi-scan (*SADABS*; Bruker, 2014 ▶)
*T*_min_, *T*_max_	0.642, 0.748	0.564, 0.746
No. of measured, independent and observed [*I* > 2(*I*)] reflections	74315, 11731, 10413	18025, 6191, 4675
*R*_int_	0.041	0.074
(sin /)_max_ (^1^)	0.879	0.695

Refinement		
*R*[*F* ^2^ > 2(*F* ^2^)], *wR*(*F* ^2^), *S*	0.039, 0.096, 1.09	0.055, 0.126, 1.01
No. of reflections	11731	6191
No. of parameters	245	255
No. of restraints	0	1
H-atom treatment	H atoms treated by a mixture of independent and constrained refinement	H atoms treated by a mixture of independent and constrained refinement
_max_, _min_ (e ^3^)	0.40, 0.30	0.72, 0.32
Absolute structure	Flack *x* determined using 4260 quotients [(*I* ^+^)(I)]/[(*I* ^+^)+(*I* )] (Parsons *et al.*, 2013 ▶)	Flack *x* determined using 1713 quotients [(*I* ^+^)(I)]/[(*I* ^+^)+(*I* )] (Parsons *et al.*, 2013 ▶)
Absolute structure parameter	0.005(12)	0.03(6)

Computer programs: *APEX2* and *SAINT* (Bruker, 2014 ▶), *SHELXS2013*, *SHELXL2014* and *SHELXTL* (Sheldrick, 2008 ▶).

## supplementary crystallographic information

### Crystal data

**Table:**

C_23_H_33_NO_2_S	*F*(000) = 420
*M**_r_* = 387.56	*D*_x_ = 1.167 Mg m^−^^3^
Monoclinic, *P*2_1_	Mo *K*α radiation, λ = 0.71073 Å
*a* = 10.535 (3) Å	Cell parameters from 4086 reflections
*b* = 7.984 (2) Å	θ = 2.2–28.7°
*c* = 13.481 (4) Å	µ = 0.16 mm^−^^1^
β = 103.519 (5)°	*T* = 100 K
*V* = 1102.5 (5) Å^3^	Needle, colorless
*Z* = 2	0.50 × 0.14 × 0.10 mm

### Data collection

**Table:**

Bruker SMART APEXII CCD platform diffractometer	4675 reflections with *I* > 2σ(*I*)
Radiation source: fine-focus sealed tube	*R*_int_ = 0.074
ω scans	θ_max_ = 29.6°, θ_min_ = 2.0°
Absorption correction: multi-scan (*SADABS*; Bruker, 2014)	*h* = −14→14
*T*_min_ = 0.564, *T*_max_ = 0.746	*k* = −11→11
18025 measured reflections	*l* = −18→18
6191 independent reflections	

### Refinement

**Table:**

Refinement on *F*^2^	Secondary atom site location: difference Fourier map
Least-squares matrix: full	Hydrogen site location: difference Fourier map
*R*[*F*^2^ > 2σ(*F*^2^)] = 0.055	H atoms treated by a mixture of independent and constrained refinement
*wR*(*F*^2^) = 0.126	*w* = 1/[σ^2^(*F*_o_^2^) + (0.0563*P*)^2^] where *P* = (*F*_o_^2^ + 2*F*_c_^2^)/3
*S* = 1.01	(Δ/σ)_max_ < 0.001
6191 reflections	Δρ_max_ = 0.72 e Å^−^^3^
255 parameters	Δρ_min_ = −0.32 e Å^−^^3^
1 restraint	Absolute structure: Flack *x* determined using 1713 quotients [(I+)-(I-)]/[(I+)+(I-)] (Parsons *et al.*, 2013)
Primary atom site location: structure-invariant direct methods	Absolute structure parameter: 0.03 (6)

### Special details

**Table:**

Geometry. All e.s.d.'s (except the e.s.d. in the dihedral angle between two l.s. planes) are estimated using the full covariance matrix. The cell e.s.d.'s are taken into account individually in the estimation of e.s.d.'s in distances, angles and torsion angles; correlations between e.s.d.'s in cell parameters are only used when they are defined by crystal symmetry. An approximate (isotropic) treatment of cell e.s.d.'s is used for estimating e.s.d.'s involving l.s. planes.
Refinement. The amine H atom was found from the difference Fourier map and refined freely. All other H atoms were placed geometrically and treated as riding atoms: methine, C—H = 1.00 Å with *U*_iso(_H) = 1.2*U*_eq_(C), methylene, C—H = 0.99 Å with *U*_iso(_H) = 1.2*U*_eq_(C), methyl, C—H = 0.98 Å with *U*_iso(_H) = 1.5*U*_eq_(C), *sp*^2^, C—H = 0.95 Å with *U*_iso(_H) = 1.2*U*_eq_(C).The absolute configuration was deterimined using 1713 quotients, which gave a Flack parameter of 0.03 (6) (Parsons and Flack, 2004, Parsons *et al.*, 2013). The value obtained without *D*_obs_(h) as a restraint was -0.04 (8), calculated from 2882 Friedel pairs (Flack, 1983).

### Fractional atomic coordinates and isotropic or equivalent isotropic displacement parameters (Å^2^)

**Table:**

	*x*	*y*	*z*	*U*_iso_*/*U*_eq_	
S1	0.14253 (7)	0.70038 (11)	0.52944 (5)	0.01937 (18)	
O1	−0.1200 (3)	0.7675 (3)	0.79000 (17)	0.0277 (6)	
O2	0.1009 (2)	0.5256 (3)	0.54810 (18)	0.0255 (5)	
N1	0.0861 (3)	0.8363 (4)	0.6010 (2)	0.0193 (6)	
H1	0.029 (4)	0.895 (5)	0.565 (3)	0.029 (11)*	
C1	0.0582 (3)	0.7907 (4)	0.7007 (2)	0.0187 (7)	
H1A	0.0758	0.6681	0.7107	0.022*	
C2	−0.0893 (4)	0.8173 (5)	0.6958 (2)	0.0228 (7)	
H2A	−0.1121	0.9380	0.6822	0.027*	
C3	−0.1748 (3)	0.7098 (6)	0.6136 (2)	0.0281 (7)	
H3A	−0.2659	0.7182	0.6187	0.042*	
H3B	−0.1462	0.5929	0.6228	0.042*	
H3C	−0.1675	0.7490	0.5463	0.042*	
C4	−0.1156 (4)	0.8932 (5)	0.8670 (3)	0.0309 (9)	
H4A	−0.0238	0.9283	0.8935	0.037*	
H4B	−0.1465	0.8434	0.9243	0.037*	
C5	−0.1973 (4)	1.0465 (5)	0.8290 (3)	0.0277 (8)	
C6	−0.1330 (4)	1.1992 (6)	0.8314 (3)	0.0399 (9)	
H6A	−0.0414	1.2050	0.8587	0.048*	
C7	−0.2017 (6)	1.3429 (6)	0.7941 (4)	0.0542 (14)	
H7A	−0.1575	1.4467	0.7949	0.065*	
C8	−0.3341 (6)	1.3334 (6)	0.7562 (3)	0.0516 (14)	
H8A	−0.3812	1.4306	0.7284	0.062*	
C9	−0.4009 (5)	1.1822 (8)	0.7580 (3)	0.0538 (14)	
H9A	−0.4931	1.1777	0.7342	0.065*	
C10	−0.3300 (4)	1.0372 (6)	0.7954 (3)	0.0375 (10)	
H10A	−0.3739	0.9337	0.7972	0.045*	
C11	0.1486 (3)	0.8807 (4)	0.7901 (2)	0.0191 (7)	
C12	0.2023 (4)	0.7877 (4)	0.8796 (2)	0.0203 (7)	
C13	0.2873 (3)	0.8671 (5)	0.9617 (2)	0.0238 (7)	
H13A	0.3229	0.8043	1.0216	0.029*	
C14	0.3208 (4)	1.0342 (5)	0.9582 (2)	0.0276 (8)	
C15	0.2652 (3)	1.1243 (5)	0.8705 (3)	0.0253 (8)	
H15A	0.2866	1.2395	0.8672	0.030*	
C16	0.1790 (3)	1.0514 (4)	0.7869 (2)	0.0196 (7)	
C17	0.1703 (4)	0.6060 (5)	0.8916 (3)	0.0264 (8)	
H17A	0.2246	0.5630	0.9557	0.040*	
H17B	0.1876	0.5416	0.8343	0.040*	
H17C	0.0780	0.5953	0.8927	0.040*	
C18	0.4126 (4)	1.1175 (6)	1.0483 (3)	0.0416 (11)	
H18A	0.4920	1.0499	1.0695	0.062*	
H18B	0.3696	1.1268	1.1051	0.062*	
H18C	0.4356	1.2295	1.0285	0.062*	
C19	0.1231 (4)	1.1646 (4)	0.6973 (2)	0.0254 (8)	
H19A	0.1332	1.2817	0.7194	0.038*	
H19B	0.0303	1.1394	0.6712	0.038*	
H19C	0.1697	1.1459	0.6433	0.038*	
C20	0.3203 (3)	0.7019 (6)	0.5841 (2)	0.0256 (7)	
C21	0.3559 (4)	0.6297 (6)	0.6915 (3)	0.0344 (9)	
H21A	0.4510	0.6197	0.7141	0.052*	
H21B	0.3160	0.5188	0.6916	0.052*	
H21C	0.3236	0.7041	0.7380	0.052*	
C22	0.3660 (4)	0.8809 (6)	0.5811 (4)	0.0463 (12)	
H22A	0.4615	0.8846	0.6018	0.069*	
H22B	0.3292	0.9493	0.6278	0.069*	
H22C	0.3368	0.9247	0.5116	0.069*	
C23	0.3749 (4)	0.5895 (7)	0.5121 (3)	0.0415 (11)	
H23A	0.4705	0.5892	0.5329	0.062*	
H23B	0.3469	0.6321	0.4422	0.062*	
H23C	0.3422	0.4751	0.5150	0.062*	

### Atomic displacement parameters (Å^2^)

**Table:**

	*U*^11^	*U*^22^	*U*^33^	*U*^12^	*U*^13^	*U*^23^
S1	0.0237 (4)	0.0206 (4)	0.0128 (3)	−0.0007 (4)	0.0020 (3)	−0.0017 (4)
O1	0.0448 (16)	0.0204 (13)	0.0200 (12)	0.0009 (11)	0.0122 (11)	0.0008 (10)
O2	0.0313 (14)	0.0179 (13)	0.0265 (12)	−0.0014 (10)	0.0053 (10)	−0.0064 (10)
N1	0.0276 (16)	0.0165 (14)	0.0120 (12)	0.0023 (12)	0.0010 (11)	0.0034 (11)
C1	0.0311 (18)	0.0125 (16)	0.0119 (13)	−0.0027 (13)	0.0042 (13)	0.0000 (12)
C2	0.0339 (19)	0.0198 (17)	0.0145 (14)	−0.0023 (14)	0.0055 (13)	0.0005 (13)
C3	0.0316 (18)	0.0297 (19)	0.0234 (15)	−0.0088 (19)	0.0072 (13)	−0.0046 (18)
C4	0.041 (2)	0.029 (2)	0.0231 (18)	0.0047 (17)	0.0093 (15)	−0.0058 (15)
C5	0.042 (2)	0.026 (2)	0.0195 (16)	0.0040 (17)	0.0152 (15)	−0.0025 (15)
C6	0.058 (2)	0.030 (2)	0.039 (2)	0.002 (2)	0.0265 (18)	−0.005 (2)
C7	0.094 (4)	0.035 (3)	0.043 (3)	0.012 (3)	0.033 (3)	0.007 (2)
C8	0.095 (4)	0.037 (3)	0.023 (2)	0.027 (3)	0.015 (2)	0.0048 (19)
C9	0.057 (3)	0.072 (4)	0.0263 (19)	0.023 (3)	−0.0033 (18)	−0.015 (2)
C10	0.041 (2)	0.045 (3)	0.0253 (19)	0.007 (2)	0.0037 (17)	−0.0105 (18)
C11	0.0244 (17)	0.0188 (17)	0.0131 (14)	0.0019 (13)	0.0025 (12)	−0.0016 (12)
C12	0.0300 (19)	0.0162 (17)	0.0155 (15)	0.0033 (14)	0.0067 (13)	0.0017 (13)
C13	0.0283 (19)	0.0253 (19)	0.0154 (15)	0.0030 (15)	0.0000 (13)	0.0037 (14)
C14	0.033 (2)	0.028 (2)	0.0179 (16)	−0.0045 (16)	−0.0009 (14)	−0.0029 (14)
C15	0.032 (2)	0.0177 (17)	0.0251 (17)	−0.0022 (15)	0.0038 (15)	−0.0023 (14)
C16	0.0262 (18)	0.0167 (17)	0.0147 (14)	0.0003 (13)	0.0023 (13)	0.0007 (12)
C17	0.040 (2)	0.0201 (18)	0.0175 (16)	0.0024 (16)	0.0044 (15)	0.0042 (14)
C18	0.049 (3)	0.039 (2)	0.027 (2)	−0.010 (2)	−0.0111 (18)	−0.0025 (19)
C19	0.040 (2)	0.0143 (19)	0.0203 (16)	−0.0027 (14)	0.0040 (14)	−0.0018 (12)
C20	0.0226 (16)	0.0331 (18)	0.0203 (14)	0.0020 (18)	0.0032 (12)	−0.0016 (19)
C21	0.029 (2)	0.051 (3)	0.0208 (17)	0.0104 (18)	0.0002 (15)	−0.0013 (17)
C22	0.027 (2)	0.041 (3)	0.068 (3)	−0.0101 (19)	0.004 (2)	0.003 (2)
C23	0.030 (2)	0.064 (3)	0.031 (2)	0.008 (2)	0.0078 (17)	−0.011 (2)

### Geometric parameters (Å, º)

**Table:**

S1—O2	1.501 (3)	C12—C13	1.402 (5)
S1—N1	1.652 (3)	C12—C17	1.507 (5)
S1—C20	1.845 (3)	C13—C14	1.384 (5)
O1—C4	1.437 (4)	C13—H13A	0.9500
O1—C2	1.437 (4)	C14—C15	1.391 (5)
N1—C1	1.487 (4)	C14—C18	1.518 (5)
N1—H1	0.82 (4)	C15—C16	1.398 (4)
C1—C11	1.529 (4)	C15—H15A	0.9500
C1—C2	1.554 (5)	C16—C19	1.513 (4)
C1—H1A	1.0000	C17—H17A	0.9800
C2—C3	1.520 (5)	C17—H17B	0.9800
C2—H2A	1.0000	C17—H17C	0.9800
C3—H3A	0.9800	C18—H18A	0.9800
C3—H3B	0.9800	C18—H18B	0.9800
C3—H3C	0.9800	C18—H18C	0.9800
C4—C5	1.515 (5)	C19—H19A	0.9800
C4—H4A	0.9900	C19—H19B	0.9800
C4—H4B	0.9900	C19—H19C	0.9800
C5—C10	1.367 (6)	C20—C22	1.511 (6)
C5—C6	1.391 (6)	C20—C21	1.522 (5)
C6—C7	1.387 (7)	C20—C23	1.529 (5)
C6—H6A	0.9500	C21—H21A	0.9800
C7—C8	1.371 (7)	C21—H21B	0.9800
C7—H7A	0.9500	C21—H21C	0.9800
C8—C9	1.400 (8)	C22—H22A	0.9800
C8—H8A	0.9500	C22—H22B	0.9800
C9—C10	1.406 (7)	C22—H22C	0.9800
C9—H9A	0.9500	C23—H23A	0.9800
C10—H10A	0.9500	C23—H23B	0.9800
C11—C16	1.403 (5)	C23—H23C	0.9800
C11—C12	1.417 (4)		
			
O2—S1—N1	110.67 (15)	C14—C13—C12	122.0 (3)
O2—S1—C20	104.37 (18)	C14—C13—H13A	119.0
N1—S1—C20	103.45 (16)	C12—C13—H13A	119.0
C4—O1—C2	118.0 (3)	C13—C14—C15	117.9 (3)
C1—N1—S1	122.8 (2)	C13—C14—C18	121.0 (3)
C1—N1—H1	113 (3)	C15—C14—C18	121.1 (4)
S1—N1—H1	110 (3)	C14—C15—C16	122.3 (3)
N1—C1—C11	112.3 (3)	C14—C15—H15A	118.8
N1—C1—C2	109.6 (3)	C16—C15—H15A	118.8
C11—C1—C2	113.7 (3)	C15—C16—C11	119.4 (3)
N1—C1—H1A	107.0	C15—C16—C19	116.9 (3)
C11—C1—H1A	107.0	C11—C16—C19	123.7 (3)
C2—C1—H1A	107.0	C12—C17—H17A	109.5
O1—C2—C3	105.7 (3)	C12—C17—H17B	109.5
O1—C2—C1	110.7 (3)	H17A—C17—H17B	109.5
C3—C2—C1	111.7 (3)	C12—C17—H17C	109.5
O1—C2—H2A	109.5	H17A—C17—H17C	109.5
C3—C2—H2A	109.5	H17B—C17—H17C	109.5
C1—C2—H2A	109.5	C14—C18—H18A	109.5
C2—C3—H3A	109.5	C14—C18—H18B	109.5
C2—C3—H3B	109.5	H18A—C18—H18B	109.5
H3A—C3—H3B	109.5	C14—C18—H18C	109.5
C2—C3—H3C	109.5	H18A—C18—H18C	109.5
H3A—C3—H3C	109.5	H18B—C18—H18C	109.5
H3B—C3—H3C	109.5	C16—C19—H19A	109.5
O1—C4—C5	113.6 (3)	C16—C19—H19B	109.5
O1—C4—H4A	108.8	H19A—C19—H19B	109.5
C5—C4—H4A	108.8	C16—C19—H19C	109.5
O1—C4—H4B	108.8	H19A—C19—H19C	109.5
C5—C4—H4B	108.8	H19B—C19—H19C	109.5
H4A—C4—H4B	107.7	C22—C20—C21	112.0 (3)
C10—C5—C6	120.7 (4)	C22—C20—C23	111.6 (4)
C10—C5—C4	121.6 (4)	C21—C20—C23	109.6 (4)
C6—C5—C4	117.7 (4)	C22—C20—S1	107.2 (3)
C7—C6—C5	120.4 (4)	C21—C20—S1	112.3 (2)
C7—C6—H6A	119.8	C23—C20—S1	103.9 (2)
C5—C6—H6A	119.8	C20—C21—H21A	109.5
C8—C7—C6	119.3 (5)	C20—C21—H21B	109.5
C8—C7—H7A	120.4	H21A—C21—H21B	109.5
C6—C7—H7A	120.4	C20—C21—H21C	109.5
C7—C8—C9	120.8 (4)	H21A—C21—H21C	109.5
C7—C8—H8A	119.6	H21B—C21—H21C	109.5
C9—C8—H8A	119.6	C20—C22—H22A	109.5
C8—C9—C10	119.3 (4)	C20—C22—H22B	109.5
C8—C9—H9A	120.3	H22A—C22—H22B	109.5
C10—C9—H9A	120.3	C20—C22—H22C	109.5
C5—C10—C9	119.4 (5)	H22A—C22—H22C	109.5
C5—C10—H10A	120.3	H22B—C22—H22C	109.5
C9—C10—H10A	120.3	C20—C23—H23A	109.5
C16—C11—C12	119.1 (3)	C20—C23—H23B	109.5
C16—C11—C1	122.5 (3)	H23A—C23—H23B	109.5
C12—C11—C1	118.4 (3)	C20—C23—H23C	109.5
C13—C12—C11	119.3 (3)	H23A—C23—H23C	109.5
C13—C12—C17	117.9 (3)	H23B—C23—H23C	109.5
C11—C12—C17	122.8 (3)		
			
O2—S1—N1—C1	27.5 (3)	C2—C1—C11—C12	98.0 (4)
C20—S1—N1—C1	−83.8 (3)	C16—C11—C12—C13	−1.7 (5)
S1—N1—C1—C11	114.3 (3)	C1—C11—C12—C13	178.9 (3)
S1—N1—C1—C2	−118.3 (3)	C16—C11—C12—C17	177.2 (3)
C4—O1—C2—C3	−146.4 (3)	C1—C11—C12—C17	−2.2 (5)
C4—O1—C2—C1	92.5 (3)	C11—C12—C13—C14	−0.1 (5)
N1—C1—C2—O1	177.6 (3)	C17—C12—C13—C14	−179.0 (4)
C11—C1—C2—O1	−55.9 (4)	C12—C13—C14—C15	1.3 (6)
N1—C1—C2—C3	60.1 (3)	C12—C13—C14—C18	179.8 (4)
C11—C1—C2—C3	−173.4 (3)	C13—C14—C15—C16	−0.7 (6)
C2—O1—C4—C5	53.4 (4)	C18—C14—C15—C16	−179.2 (4)
O1—C4—C5—C10	63.7 (5)	C14—C15—C16—C11	−1.2 (5)
O1—C4—C5—C6	−117.6 (4)	C14—C15—C16—C19	178.6 (3)
C10—C5—C6—C7	−3.7 (6)	C12—C11—C16—C15	2.3 (5)
C4—C5—C6—C7	177.6 (3)	C1—C11—C16—C15	−178.3 (3)
C5—C6—C7—C8	0.9 (6)	C12—C11—C16—C19	−177.4 (3)
C6—C7—C8—C9	2.2 (7)	C1—C11—C16—C19	2.0 (5)
C7—C8—C9—C10	−2.6 (6)	O2—S1—C20—C22	−172.3 (3)
C6—C5—C10—C9	3.3 (5)	N1—S1—C20—C22	−56.5 (3)
C4—C5—C10—C9	−178.0 (3)	O2—S1—C20—C21	−48.9 (3)
C8—C9—C10—C5	−0.2 (6)	N1—S1—C20—C21	66.9 (3)
N1—C1—C11—C16	43.8 (4)	O2—S1—C20—C23	69.4 (3)
C2—C1—C11—C16	−81.3 (4)	N1—S1—C20—C23	−174.7 (3)
N1—C1—C11—C12	−136.8 (3)		

### Hydrogen-bond geometry (Å, º)

**Table:**

*D*—H···*A*	*D*—H	H···*A*	*D*···*A*	*D*—H···*A*
N1—H1···O2^i^	0.83 (4)	2.08 (4)	2.890 (4)	169 (4)
C7—H7*A*···O1^ii^	0.95	2.59	3.501 (6)	160

Symmetry codes: (i) −*x*, *y*+1/2, −*z*+1; (ii) *x*, *y*+1, *z*.
